# A tendency toward evening chronotype associates with less healthy diet among preschoolers: cross-sectional findings from the DAGIS study

**DOI:** 10.1093/sleepadvances/zpae026

**Published:** 2024-04-20

**Authors:** Anna M Abdollahi, Xinyue Li, Ilona Merikanto, Henna Vepsäläinen, Reetta Lehto, Jenna Rahkola, Kaija Nissinen, Noora Kanerva, Eva Roos, Maijaliisa Erkkola

**Affiliations:** Department of Food and Nutrition, University of Helsinki, Helsinki, Finland; School of Data Science, City University of Hong Kong, Hong Kong SAR, China; Faculty of Medicine, University of Helsinki, Helsinki, Finland; Department of Public Health and Welfare, Finnish Institute for Health and Welfare, Helsinki, Finland; Orton Orthopaedics Hospital, Helsinki, Finland; Department of Food and Nutrition, University of Helsinki, Helsinki, Finland; Department of Food and Nutrition, University of Helsinki, Helsinki, Finland; Folkhälsan Research Center, Folkhälsan, Helsinki, Finland; Folkhälsan Research Center, Folkhälsan, Helsinki, Finland; Department of Food and Nutrition, University of Helsinki, Helsinki, Finland; School of Food and Agriculture, Seinäjoki University of Applied Sciences, Seinäjoki, Finland; Department of Food and Nutrition, University of Helsinki, Helsinki, Finland; Folkhälsan Research Center, Folkhälsan, Helsinki, Finland; Department of Food Studies, Nutrition and Dietetics, Uppsala University, Uppsala, Sweden; Department of Public Health, University of Helsinki, Helsinki, Finland; Department of Food and Nutrition, University of Helsinki, Helsinki, Finland

**Keywords:** circadian misalignment, sleep–wake rhythm, irregular sleep, added sugar intake, fruit and vegetable consumption

## Abstract

**Study Objectives:**

Evidence suggests that adolescents and adults with a later chronotype have poorer sleep habits and are more susceptible to unhealthy behaviors, but little is known about these associations in younger children. The objective of the study was to (1) identify and compare individual chronotype tendencies among preschool-aged children and (2) investigate associations of sleep dimensions and chronotype with diet.

**Methods:**

Participants were 636 3–6 years old (mean ± SD age: 4.74 ± 0.89 years, 49% girls) preschoolers from the cross-sectional Increased Health and Well-Being in Preschoolers (DAGIS) study in Finland. Sleep duration, sleep variability (in duration and midpoint), social jetlag, and midsleep on weekends adjusted for sleep debt (MSWEadj) were measured with 7-day actigraphy. Morning, intermediate, and evening chronotype tendencies were defined based on the lowest and highest 10th percentile cutoffs of MSWEadj. Food, energy, and macronutrient intake were assessed from 3-day records. Associations between sleep dimensions and diet were assessed with regression models.

**Results:**

MSWEadj was 1:13 ± 14 minutes for morning (*n* = 64), 2:25 ± 28 minutes for intermediate (*n* = 560), and 3:38 ± 15 minutes for evening (*n* = 64) chronotype tendency. Children with an evening chronotype tendency had greater social jetlag and sleep variability. Having an evening chronotype tendency was associated with higher added sugar, higher sugary food consumption, and lower vegetable consumption compared to intermediate tendency types. A later chronotype (MSWEadj) was associated with higher sugary food consumption, as well as lower vegetable and fiber intake. Sleep duration, social jetlag, and sleep variability were not associated with diet.

**Conclusions:**

Several less healthy sleep and diet behaviors were observed among children with later chronotypes. Future public health interventions aimed towards children would benefit from taking into account chronotype.

**Statement of Significance**

Poor sleep habits and a later chronotype are believed to be associated with many health-related outcomes and unhealthy behaviors, including poorer diet, among adolescents and adults. However, little is known about such associations among young children. In a cross-sectional study of preschoolers, we found children with an evening chronotype tendency already exhibited greater social jetlag (i.e. weekend catch-up sleep) and day-to-day variability in sleep. An evening chronotype tendency was associated with less healthy diets (reported by caretakers) higher in added sugar and lower vegetable consumption, compared to intermediate-type children after adjustment for sociodemographic factors, daylight, sleep duration, and physical activity. Our findings suggest that circadian misalignment, stemming from a mismatch in internal (i.e. chronotype) and environmental (i.e. societal) schedules, may begin at a worrisomely young age, and children with an evening chronotype tendency may be more prone to less healthy sleep and dietary behaviors.

The role of habitual sleep during childhood has gained traction in research regarding the sleep-obesity nexus. Shorter sleep duration is a well-established risk factor for obesity in school-aged children, but research concerning different sleep dimensions (i.e. variability, timing, and chronotype) with health behaviors related to weight status is limited [1]. Considering that early childhood is a period with rapid biological developments where lasting dietary habits are formed [2], it is especially important to better understand associations between sleep dimensions and diet during early childhood.

Chronotype reflects an individual’s intrinsic circadian time in physiological functions and behavior, though its phenotypical expression naturally shifts throughout the lifecycle, with children typically exhibiting an earlier chronotype and adolescents a later chronotype [3–5]. Despite the prominence of earlier chronotypes in childhood, having an evening chronotype could be as prevalent as 10%–26% among preschool-aged children [6–9]. Since children are often subject to environmental (e.g. parental and societal) schedules that affect their sleep–wake behaviors, circadian misalignment can potentially begin to accumulate after the start of preschool, especially in later chronotypes whose internal clocks do not align with societal schedules [10]. Circadian misalignment can result from the desynchronization of internal circadian time with sleep–wake behaviors, which has shown to disrupt metabolic and appetite-related hormonal balance in adolescents and adults [11–13]. Chronic circadian misalignment can be caused by chronotype, differences during weekday and weekend sleep (social jetlag), and sleep-related disorders [11].

Chronic circadian misalignment throughout childhood may contribute to the associations observed between poor sleep habits and increased adiposity in older children [14, 15]. Evening chronotype adolescents and adults, who often need to conform their habits to fit societal norms, are particularly at risk for poor sleep habits and chronic circadian misalignment [13, 16]. As expected, poorer diet quality and increased adiposity have been associated with an evening chronotype in both adolescents and adults [14, 16–19]. Acute circadian misalignment and sleep deprivation from intervention studies among adolescents and adults have similarly resulted in higher intake of energy and less healthy food choices, particularly with sweets [20, 21]. Still, very few studies have examined the associations of habitual sleep parameters outside of sleep duration, especially measures indicative of circadian misalignment, with overall dietary intake in early childhood.

Short sleep duration has been consistently associated with less healthy diet habits among children, though there are inconclusive findings for various dietary outcomes, such as fruit, vegetable, sweets, and sugar-sweetened beverage (SSB) consumption, as well as energy and macronutrient intake [22, 23]. Despite the historical emphasis on studying sleep duration, a systematic review of sleep and diet in children under five still concluded that more studies are needed among preschool-aged children to examine the potential associations between shorter sleep duration and poorer diet quality [22]. Fewer studies have examined sleep dimensions related to circadian misalignment, such as later chronotype, variability in sleep habits, and social jetlag. For instance, findings from adolescents (11–17 years old) and adults suggest that associations exist between later chronotype and less healthy diet habits [24–26], yet no studies have explored chronotype and dietary intake in early childhood. Likewise, irregularity of circadian timing (i.e. day-to-day variability in sleep duration or timing) has been associated with increased obesity and poorer dietary habits in adolescents and adults [27–29], but has been scarcely explored in association with diet among younger children. One study on 2- to 6-year-old children predisposed to obesity found greater sleep variability to be associated with greater added sugar and SSB intake [30]. Lastly, social jetlag has been identified as a risk factor for metabolic disturbance in children aged 4–10 years [31] and has been associated with increased energy intake [32] and unfavorable diet patterns in preschoolers [33]. Given the evidence surrounding the importance of sleep and health, it is crucial for studies to include various sleep dimensions in order to better understand how sleep and circadian timing are associated with overall diet in early life and across the lifespan.

To the best of our knowledge, this is the first study to investigate the associations of various sleep dimensions, including chronotype, duration, variability in duration and timing, and social jetlag, together with dietary intake among preschool-aged children. Since varying aspects of dietary intake have been studied in relation to sleep [22–30, 32, 33], this study comprehensively investigates diet as food groups, energy, and nutrient intake. The first objective of this study is to describe sociodemographic and behavioral characteristics of morning and evening chronotype tendencies in 3- to 6-year-old children in Finland. We hypothesize that an evening chronotype tendency will have more unfavorable behavioral characteristics compared to the intermediate and morning tendency types. The second objective is to examine the associations between the sleep dimensions with diet, including vegetable, fruit, and sugary food and beverage intake, as well as fiber, added sugar, total energy, and macronutrient intake, which are reflective of diet quality. We hypothesize that the children with a later chronotype, shorter sleep duration, higher variability in duration and timing, and greater social jetlag will have higher energy intake and poorer dietary habits.

## Materials and Methods

### Study population

The study population consisted of participants from the Increased Health and Well-being in Preschoolers (DAGIS) study, which investigates the energy-balance-related behaviors among preschool-aged children in Finland [34]. This study is based on the cross-sectional DAGIS Survey from September 2015 to April 2016 among 66 preschools throughout southern and western Finland. Parents or legal guardians provided consent for themselves and their children to participate in the study. A total of 864 children (24% of those invited) participated in the study. Of those, 636 (74%) were eligible for the current study based on available sleep and diet data. The University of Helsinki Ethical Review Board in the Humanities and Social and Behavioral Sciences approved the study in 2/2015 (#6/2015).

### Sleep assessment

Sleep was assessed with a hip-worn ActiGraph wGT3X-BT activity monitor (Pensacola, FL, USA) recorded at 30 Hz with the idle sleep mode enabled to preserve energy. Participants and families were instructed to have the participants wear the accelerometer for an assigned consecutive 7-day period and to remove the device only for water-based activities. A valid night was considered at least 16 hours of wear time defined by GGIRv2.5 from a noon–noon window using the default 5-second epoch level [35]. Actigraphy data were then processed by an open-sourced, data-driven Hidden Markov Model (HMM) algorithm (R-package “hmmacc”), which characterizes sleep–wake epochs and defines nighttime sleep onset and wake up based on unique activity profiles of individuals instead of population-specific thresholds. Therefore, the unsupervised model does not rely on sleep diaries and is not specific to an age group or device [36]. Additional details on the algorithm, which has been validated against polysomnography [36], have been previously described elsewhere [37]. Nevertheless, parent-reported sleep and the HMM algorithm nighttime sleep–wake behaviors have been found to be comparable within the DAGIS study population in a previous study [37]. To account for possible misclassified non-wear time during the idle sleep mode, all non-wear time lasting <2 hours between sleep onset and wake-up time were ignored. Additionally, daytime sleep was not accounted for in this study. To best assess presumed free-day sleep (days when wake-up is unscheduled), weekend nights were utilized, which were defined as Friday–Saturday and Saturday–Sunday nights. Sleep data were considered adequate when there was a minimum of five nights, of which two were weekend nights. Children with inadequate sleep data were excluded (*n* = 175, 20%). Nevertheless, the majority (93%, *n* = 592) of the analytical study sample (*n* = 636) had the full seven nights of sleep data.

Sleep variables (duration, variability, social jetlag, and chronotype) were calculated from sleep onset and wake-up times. Sleep onset, wake-up, and duration refer to the nighttime sleep period and are presented as weighted means calculated as: (weekday night mean × 5 + weekend night mean × 2)/7. To evaluate within-individual variability, the standard deviations of sleep midpoint (referred to as variability in timing) and duration (referred to as variability in duration) were averaged and similarly weighted. The midpoint of sleep was calculated as half of the sleep duration since sleep onset and was calculated separately for weekday and weekend nights. Social jetlag was defined as the difference in weekend to weekday average sleep midpoint. Chronotype was estimated as both a continuous and a categorical variable, calculated based on the Munich ChronoType Questionnaire estimation of chronotype [3] with the assumption that sleep on weekend nights was free from a set schedule, since no child attended preschool on weekends. The weekend sleep midpoint adjusted for possible sleep debt accumulated on weekday nights (MSWEadj) [3] was used as a continuous variable, calculated as follows: MSWE − (weekend sleep duration − average weekly sleep duration)/2. In cases where sleep duration on weekdays was not less than on weekends (i.e. no sleep debt accumulated on weekday nights), the value for MSWE was simply used to represent MSWEadj.

An explorative approach was used to indicate chronotype as a categorical variable from MSWEadj conservatively based on previous reports on the prevalence of evening chronotype among similarly aged children (10%–26%) [6–9]. Children in the lowest 10th percentiles of the MSWEadj were referred to as having a morning chronotype tendency, the highest 10th percentile as having an evening chronotype tendency, and in between the 10th–90th percentiles as having an intermediate chronotype tendency.

### Dietary assessmient

A 3-day food diary was recorded by parents at home and staff in the preschools. Details of the process have been previously described elsewhere [38]. In brief, 2 weekday days and 1 weekend day were assigned to be collected. The preschool staff were given a separate pre-coded food record with the dates matching with the home record and with predefined sections for the meals that were offered for children in preschool. In Finland, meals in preschools are provided meals free of charge. A child’s food portion size estimation booklet was distributed to parents and preschool staff to aid in accurate assessment of intake [39]. Trained nutrition researchers verified the completeness of records, contacted the recorder for missing details when necessary, and calculated the reported dietary intake via AivoDiet dietary software 2.2.0.0 (Mashie FoodTech Solutions Finland Oy, Turku, Finland). Of the 636 participants, those without dietary data for 3 days were excluded (*n* = 53, 6%).

Food variables were chosen as food groups that reflect dietary habits. Food groups were assessed as grams per megajoule (g/MJ) and included vegetables (i.e. fresh, frozen, or canned vegetables, excluding potatoes and vegetables incorporated into mixed dishes), fresh fruit, sugar-sweetened foods (i.e. pastries, cakes, cookies, doughnuts, candies, chocolates, puddings, ice cream, and other dairy-based desserts), and SSBs (i.e. juices and sodas with added sugar). Energy and nutrient variables included total daily energy (kJ), percentage of carbohydrate, protein, fat, and added sugar intake compared to total energy intake (%E) and fiber (g/MJ).

### Sociodemographic and additional behavioral variables

Sociodemographic and behavioral variables previously identified as potential confounders were included in the descriptive table (Table 1) [37, 40]. Parents reported the child’s birth date and sex, number of adults living in the household, and parental education level(s). The highest familial parental education was divided into low (i.e. high school or vocational diploma or less), medium (i.e. associate or bachelor’s degree), and high (i.e. master’s, licentiate or doctoral degree) categories. Children’s screen time (min/day) was assessed from parent-reported TV, computer, tablet, and phone use in a sedentary behavior diary for the same 7-day period as the children were instructed to wear the actigraphy monitors [41]. Light physical activity, moderate-to-vigorous physical activity (MVPA), and sedentary time were assessed as minutes per day with Butte et al. childhood activity intensity thresholds from actigraphy data [42, 43]. Children’s height, weight, and waist circumference were measured by trained researchers and described in detail elsewhere [43]. Body mass index (BMI), calculated as weight (kg)/height^2^ (m), was adjusted for age and sex according to Finnish growth standards and converted to correspond to adult BMI (ISO-BMI) [44]. Outdoor light exposure is considered the strongest environmental zeitgeber (i.e. an exogenous factor able to influence the circadian clock) affecting the sleep–wake cycle [40], and seasonal variation of sunlight is considerable in countries in high latitudes, like Finland. Most (43%) participants were surveyed in the fall months between September and October, 36% in the winter months between November and December, and 21% in the spring between January and March. In order to best adjust for available outdoor light, the average (hours/day) length of daylight (sunrise to sunset) in Helsinki, Finland was calculated for days with sleep data using the R package “suncalc” version 0.5.1.

**Table 1.** Descriptive Table of Study Participants Overall and Grouped by Chronotype Tendency Categorized From MSWEadj^1^

	Overall (*n* = 636)	Morning tendency (*n* = 64)	Intermediate tendency (*n* = 508)	Evening tendency (*n* = 64)	*P*-value^2^
	Mean ± SD, *n*(%)	Mean ± SD, *n*(%)	Mean ± SD, *n*(%)	Mean ± SD, *n*(%)	
Age (years)	4.74 ± 0.89	4.54 ± 0.94	4.75 ± 0.88	4.85 ± 0.90	.12
Sex (female)	309 (48%)	25 (39%)	247 (49%)	37 (58%)	.11
ISO-BMI^3^	21.9 ± 3.6	21.8 ± 3.8	22.0 ± 3.6	21.3 ± 3.1	.37
Waist circumference (cm)	53.3 ± 9.7	53.6 ± 4.3	53.3 ± 10.6	53.7 ± 4.2	.92
Parental education level^4^					.17
Low	130 (20%)	16 (25%)	101 (20%)	13 (20%)	
Medium	270 (42%)	33 (52%)	208 (41%)	29 (45%)	
High	234 (37%)	15 (23%)	198 (39%)	21 (35%)	
Single adult household	44 (7%)	9 (14%)	29 (6%)	6 (9%)	**.04**
Moderate-to-vigorous physical activity (min/day)	72.5 ± 22.1	73.2 ± 22.4	72.7 ± 22.0	70.3 ± 22.3	.70
Light physical activity (min/day)	466 ± 39	455 ± 43	466 ± 39	477 ± 37	**<.01**
Sedentary time (min/day)	401 ± 46	404 ± 51	402 ± 46	392 ± 44	.24
Screen time (min/day)	74.2 ± 35.4	68.8 ± 34.7	73.8 ± 34.7	82.6 ± 40.1	.08
Daylight length (h:mm)	9:31 ± 2:18	9:51 ± 2:28	9:35 ± 2:17	8:49 ± 2:07	**.02**
*Sleep variables (hh:mm)*					
MSWEadj	2:25 ± 00:41	1:13 ± 00:14	2:25 ± 00:28	3:38 ± 00:15	**<.001**
Duration	9:43 ± 00:31	9:54 ± 00:30	9:43 ± 00:31	9:36 ± 00:29	**<.001**
Onset time	21:16 ± 00:38	20:16 ± 00:28	21:21 ± 00:29	22:09 ± 00:26	**<.001**
Wake-up time	6:58 ± 00:32	6:16 ± 00:26	6:58 ± 00:26	7:45 ± 00:22	**<.001**
Social jetlag (min)	26.5 ± 27.1	-3.1 ± 18.8	26.2 ± 24.1	58.0 ± 22.7	**<.001**
Variability in duration (min)	40.3 ± 16.8	34.3 ± 14.5	40.4 ± 16.7	46.1 ± 18.5	**<.001**
Variability in onset (min)	29.9 ± 13.7	25.6 ± 14.1	29.7 ± 13.2	36.4 ± 15.1	**<.001**
Variability in midpoint (min)	25.4 ± 10.7	19.9 ± 9.0	24.8 ± 10.1	36.5 ± 10.5	**<.001**
*Food consumption (g/MJ)*					
Vegetables^5^	12.9 ± 8.2	14.1 ± 8.4	13.0 ± 8.2	10.7 ± 7.3	**.04**
Fresh fruits	18.4 ± 14.0	19.2 ± 14.6	18.0 ± 13.9	20.5 ± 13.9	.37
Sugar-sweetened foods^6^	10.7 ± 8.5	8.4 ± 6.6	10.4 ± 8.1	14.9 ± 11.4	**<.001**
Sugar-sweetened beverages^7^	11.3 ± 13.0	10.6 ± 12.3	11.0 ± 12.9	14.3 ± 14.6	.15
*Energy intake*					
MJ/day	5.80 ± 1.12	5.76 ± 1.14	5.83 ± 1.12	5.58 ± 1.07	.22
*Nutrient intake (%E)*					
Carbohydrate	48.3 ± 4.8	49.0 ± 5.0	48.1 ± 4.8	49.0 ± 4.5	.19
Protein	16.2 ± 2.3	16.3 ± 2.3	16.3 ± 2.1	15.7 ± 3.0	.15
Fat, total	32.5 ± 4.6	31.6 ± 4.6	32.6 ± 4.7	32.4 ± 4.0	.30
Saturated fatty acid	12.4 ± 2.6	12.0 ± 2.4	12.4 ± 2.6	12.4 ± 2.2	.50
Monounsaturated fatty acid	11.0 ± 1.9	10.8 ± 1.9	11.1 ± 1.9	11.0 ± 1.7	.53
Polyunsaturated fatty acid	5.05 ± 1.23	4.99 ± 1.18	5.07 ± 1.24	4.95 ± 1.18	.73
Added sugar	9.14 ± 4.10	9.04 ± 4.52	8.98 ± 4.00	10.50 ± 4.25	**.02**
Fiber (g/MJ)	2.44 ± 0.61	2.45 ± 0.53	2.45 ± 0.62	2.32 ± 0.52	.25

BMI, body mass index; MSWEadj, midpoint of sleep on weekend, adjusted for possible sleep debt from weekdays; %E, percentage from total energy intake.

^1^Morning tendency = Earliest 10th MSWEadj percentile, Intermediate tendency = 10–90th MSWEadj percentile, Evening tendency = Latest 10th MSWEadj percentile.

^2^Exact *p*-value of ANOVA for continuous variables and chi-squared test for categorical variables (post hoc within group differences are described in the text). Bolded values indicate statistical significance (p<0.05).

^3^Body mass index adjusted for age and sex (ISO-BMI).

^4^Low = high school or vocational diploma or less, medium = associate’s or bachelor’s degree, high = master’s, licentiate, or doctoral degree.

^5^Vegetables include fresh, frozen, or canned vegetables, excluding potatoes and vegetables from mixed dishes.

^6^Sugar-sweetened foods include pastries, cakes, cookies, doughnuts, candies, chocolate, puddings, ice cream, and other dairy-based desserts.

^7^Sugar-sweetened beverages include all juices and sodas with added sugar, excluding 100% fruit juices and artificially sweetened beverages.

### Statistical analysis

Differences in descriptive variables between chronotype categories were detected with analysis of variance for continuous variables and chi-squared tests for categorical variables. Statistically significant differences between chronotype groups were further examined with Tukey–Kramer’s post hoc test. Food variables were non-normally distributed and, therefore, square-root transformed for regression analyses. Non-transformed results are reported within text when statistical significance is similar. In the case of SSBs, where the number of non-users was high (*n* = 228, 36%), a constant value was added to the variable prior to transformation. Multivariate linear regression was performed to assess the associations between sleep dimensions (i.e. MSWEadj, chronotype tendency, sleep duration, variability in duration and timing, and social jetlag) and diet (i.e. food group, energy, and nutrient intakes). For models with chronotype tendency, intermediate tendency type was used as the reference category. Model covariates were determined from the theory-based confounders with assessment of collinearity and correlation with outcome and predictor variables. Crude models are presented in Supplementary Tables. Model 1 included adjustment for age and sex, as well as sleep duration for chronotype. Model 2 included variables from model 1 and ISO-BMI, length of daylight, living in a single adult household, light physical activity, and MVPA. Parental education level was added to the model as a proxy for socioeconomic status without significant change in results and was therefore left out of the final adjusted models. A *p*-value <.05 was used to determine statistical significance. To reduce the risk of Type I error in multiple comparisons, Benjamini and Hochberg adjusted *p*-values were used in all regression models. Statistical software R version 4.1.2 was used for statistical analysis.

## Results

### Descriptives of the study population by chronotype tendency

#### Chronotype tendency and sociodemographic and behavioral variables.

The distribution of sociodemographic factors did not differ among the morning (*n* = 64), intermediate (*n* = 508), or evening (*n* = 64) chronotype tendency groups, with the exception of single adult households, with post hoc analysis revealing morning tendency having a slightly higher frequency than intermediate types (*p* = .045). Physical activity levels only differed in daily light physical activity, which was 22.9 minutes (*p* < .01) lower among morning tendency types than evening tendency. On average, the length of available daylight was shorter (i.e. the measurement date was closer to winter equinox) when children with evening chronotype tendency wore the actigraphy device than children with morning (46 minutes, *p* = .03) and intermediate (62 minutes, *p* = .03) tendency.

Compared to the study population, children excluded from the final sample size (*n* = 228 out of 864) had lower socioeconomic status as represented by parental education levels, but were similar in all other sociodemographic and behavioral characteristics.

#### Chronotype tendency and sleep dimensions.

The mean MSWEadj (chronotype assessed as a continuous variable) for participants differed by over an hour between morning, intermediate, and evening chronotype tendencies (Table 1). Specifically, children who were classified as a morning tendency chronotype had MSWEadj 72 minutes (*p* < .001) and 145 minutes (*p* < .001) earlier than the intermediate and evening chronotype, respectively. Evening chronotype tendency had 73 minutes (*p* < .001) later MSWEadj than intermediate tendency.

Sleep duration, timing, variability, and social jetlag all significantly varied among different chronotype tendencies (Table 1). Children with a morning chronotype tendency had longer sleep duration than both intermediate (estimated difference: 11 minutes, *p* = .01) and evening tendency types (18 minutes, *p* < .01). Social jetlag and variability in sleep duration differed significantly between all chronotype tendency types, with morning tendency having an estimated 61 minutes less social jetlag (*p* < .001) and 12 minutes (*p* < .001) less variation in sleep duration on average than children in the evening tendency chronotype. Children with evening tendency types had more variability in sleep midpoint than morning (16.6 minutes, *p* < .001) and intermediate tendency types (11.7 minutes, *p* < .001).

#### Chronotype tendency and diet.

Vegetable, sugar-sweetened foods, and added sugar intake significantly varied among different chronotype tendencies (Table 1). Specifically, children with an evening chronotype tendency consumed less vegetables than children with morning chronotype tendency (3.44 MJ/day, *p* = .04), but not intermediate chronotype tendency (*p* = .08). Though no statistically significant differences were seen between early and intermediate tendency types on sugary food consumption in post hoc comparisons, children with evening tendency consumed more sugary foods on average than both early (4.53 g/MJ, *p* < .001) and intermediate (6.55 g/MJ, *p* < .001) tendency types. Children with evening tendencies had higher intake of added sugar than intermediate tendency chronotypes (1.57 %E, *p* < .01), but not morning tendency type children (*p* = .09).

### Associations of sleep dimensions and chronotype tendency with food consumption and energy and nutrient intake.

#### Chronotype as a continuous variable MSWEadj.

Chronotype as assessed by the MSWEadj continuous variable was associated with reported vegetable and sugary food consumption (Table 2), as well as fiber intake (Table 3). Every hour increase in MSWEadj was associated with lower vegetable consumption (non-transformed B-estimate: −1.35 g/MJ, 95% CI: (−2.38, −0.33), *p *< .01) and increased sugary food consumption (non-transformed B-estimate: 2.54 g/MJ, 95% CI: [1.50, 3.58], *p* < .0001; Table 2). A later MSWEadj was associated with lower fiber (B-estimate: −0.09 g/MJ, 95% CI: [−0.17, −0.02], *p* = .01) intake (Table 3). Approximately 2.2%–5.1% of the variance in dietary intake was explained by all fully adjusted models. Chronotype assessed as either continuous MSWEadj or tendency type was not associated with saturated, polyunsaturated, or monounsaturated fat intake (Supplemental Table 1).

**Table 2.** Multivariate Linear Regression Analyses of Sleep and Chronotype Tendency as Predictors of Food Consumption in DAGIS Study Participants (*n* = 636)

	Vegetable^1^ (g/MJ)		Fruit^1^ (g/MJ)		Sugary foods^1^ (g/MJ)		Sugar-sweetened beverages^1^ (g/MJ)	
	Model 1 B (95% CI)	Model 2 B (95% CI)	Model 1 B (95% CI)	Model 2 B (95% CI)	Model 1 B (95% CI)	Model 2 B (95% CI)	Model 1 B (95% CI)	Model 2 B (95% CI)
MSWEadj	−0.17* *(−0.31, −0.04)*	−0.20* *(−0.35, −0.06)*	0.01 (−0.19, 0.21)	0.06 (−0.16, 0.27)	0.35*** *(0.20, 0.51)*	0.37*** *(0.21, 0.53)*	0.19 (−0.02, 0.39)	0.11 (−0.12, 0.33)
*Chronotype tendency* ^2^								
Intermediate	ref	ref	ref	ref	ref	ref	ref	ref
Morning	0.18 (−0.13, 0.49)	0.19 (−0.14, 0.52)	0.02 (−0.43, 0.48)	−0.13 (−0.62, 0.36)	−0.38 (−0.73, −0.04)	−0.44 (−0.81, −0.06)	−0.05 (−0.53, 0.43)	−0.02 (−0.53, 0.49)
Evening	−0.38* *(−0.68, −0.08)*	−0.41* *(−0.74, −0.08)*	0.31 (−0.14, 0.76)	0.33 (−0.15, 0.81)	0.60** *(0.26, 0.95)*	0.58** *(0.21, 0.94)*	0.42 (−0.05, 0.90)	0.20 (−0.30, 0.71)
Sleep duration	0.13 (−0.04, 0.31)	0.11 (0.10, 0.33)	0.21 (−0.05, 0.47)	0.05 (−0.26, 0.37)	−0.07 (−0.27, 0.13)	0.13 (−0.11, 0.37)	−0.0004 (−0.27, 0.27)	0.08 (−0.25, 0.41)
Social jetlag	−0.11 (−0.31, 0.09)	−0.08 (−0.31, 0.14)	−0.08 (−0.38, 0.22)	0.01 (−0.32, 0.33)	0.21 (−0.02, 0.44)	0.23 (−0.02, 0.48)	0.17 (−0.15, 0.49)	0.09 (−0.25, 0.43)
Variability in sleep duration	−0.16 (−0.48, 0.17)	−0.14 (−0.51, 0.23)	0.32 (−0.16, 0.80)	0.15 (−0.39, 0.69)	0.04 (−0.33, 0.42)	0.19 (−0.22, 0.61)	0.25 (−0.26, 0.76)	0.26 (−0.31, 0.82)
Variability in sleep midpoint	−0.23 (−0.74, 0.27)	−0.16 (−0.73, 0.42)	0.04 (−0.72, 0.79)	0.13 (−0.96, 0.71)	0.22 (−0.36, 0.81)	0.42 (−0.23, 1.06)	0.45 (−0.34, 1.25)	0.38 (−0.49, 1.25)

^1^Variables square-root transformed.

^2^Morning tendency = Earliest 10th MSWEadj percentile, Intermediate tendency = 10–90th MSWEadj percentile, Evening tendency = Latest 10th MSWEadj percentile. MSWEadj = Midsleep on weekends, adjusted for possible sleep debt on weekdays. Model 1—adjusted for age and sex (and sleep duration for MSWEadj and chronotype tendency). Model 2—adjusted for variables in Model 1 + age and sex-adjusted body mass index, daylight length, single adult household, and physical activity.

Values in italics indicate significance with Benjamini and Hochberg adjusted *p*-values and * indicates the level of significance (* = adj.*p* < .05, ** = adj.*p* < .01, *** = adj.*p* < .001).

**Table 3.** Multivariate Linear Regression Analyses of Sleep and Chronotype Tendency as Predictors of Energy and Nutrient Intake in DAGIS Study Participants (*n* = 636)

	Energy (MJ)		Carbohydrate (%E)		Protein (%E)		Fat (%E)		Added sugar (%E)		Fiber (g/MJ)	
	Model 1 B (95% CI)	Model 2 B (95% CI)	Model 1 B (95% CI)	Model 2 B (95% CI)	Model 1 B (95% CI)	Model 2 B (95% CI)	Model 1 B (95% CI)	Model 2 B (95% CI)	Model 1 B (95% CI)	Model 2 B (95% CI)	Model 1 B (95% CI)	Model 2 B (95% CI)
MSWEadj	−0.18* *(−0.30, −0.06)*	−0.11 (−0.23, 0.01)	0.03 (−0.58, 0.53)	−0.11 (−0.49, 0.71)	−0.08 (−0.34, 0.18)	−0.09 (−0.37, 0.19)	0.19 (−0.34, 0.73)	0.05 (−0.53, 0.63)	0.50 (0.03, 0.97)	0.55 (0.05, 1.05)	−0.09* *(−0.16, −0.02)*	−0.09* *(−0.17, −0.02)*
Chronotype tendency^1^												
Intermediate	ref	ref	ref	ref	ref	ref	ref	ref	ref	ref	ref	ref
Morning	0.01 (−0.28, 0.26)	0.07 (−0.21, 0.34)	0.82 (−0.45, 2.08)	0.60 (−0.76, 1.96)	−0.05 (−0.64, 0.54)	−0.02 (−0.62, 0.66)	−0.85 (−2.07, 0.36)	−0.66 (−1.98, 0.66)	−0.13 (−0.94, 1.21)	−0.05 (−1.08, 1.18)	−0.02 (−0.18, 0.14)	0.05 (−0.22, 0.11)
Evening	−0.27 (−0.53, 0.002)	−0.05 (−0.32, 0.22)	0.90 (−0.36, 2.16)	0.62 (−0.72, 1.95)	−0.55 (−1.13, 0.04)	−0.50 (−1.13, 0.12)	−0.21 (−1.42, 1.00)	−0.01 (−1.28, 1.31)	1.53* (0.46, 2.60)	1.43* *(0.32, 2.54)*	−0.13 (−0.29, 0.03)	−0.16 (−0.33, 0.001)
Sleep duration	−0.02 (−0.17, 0.14)	−0.07 (−0.25, 0.10)	0.19 (−0.53, 0.91)	0.66 (−0.21, 1.54)	0.08 (−0.42, 0.26)	−0.40 (−0.81, 0.01)	−0.19 (−0.89, 0.50)	−0.34 (−1.18, 0.51)	−0.08 (−0.70, 0.53)	0.25 (−0.48, 0.98)	0.06 (−0.03, 0.15)	0.08 (−0.03, 0.18)
Social jetlag	−0.15 (−0.32, 0.03)	−0.08 (−0.26, 0.10)	−0.16 (−1.00, 0.68)	0.27 (−0.66, 1.15)	0.09 (−0.30, 0.48)	0.01 (−0.41, 0.44)	0.13 (−0.67, 0.94)	−0.24 (−1.12, 0.63)	0.10 (−0.62, 0.81)	0.36 (−0.40, 1.11)	−0.07 (−0.18, 0.03)	−0.08 (−0.19, 0.03)
Variability in sleep duration	−0.29 (−0.58, −0.01)	−0.01 (−0.29, 0.31)	−0.79 (−2.14, 0.55)	−0.61 (−2.11, 0.88)	−0.47 (−1.10, 0.15)	−0.65 (−1.35, 0.05)	1.48 (0.19, 2.76)	1.42 (−0.03, 2.86)	0.23 (−0.92, 1.37)	0.34 (−0.91, 1.57)	−0.04 (−0.20, 0.13)	−0.04 (−0.22, 0.15)
Variability in sleep midpoint	−0.56 (−1.01, −0.12)	−0.13 (−0.60, 0.34)	−0.21 (−2.31, 1.89)	−0.11 (−2.43, 2.21)	−0.41 (−1.38, 0.57)	−0.33 (−1.42, 0.75)	0.97 (−1.05, 2.99)	0.80 (−1.45, 3.04)	1.16 (−0.63, 2.95)	2.14 (0.22, 4.07)	−0.20 (−0.47, 0.06)	−0.29 (−0.58, 0.01)

^1^Morning tendency = Earliest 10th MSWEadj percentile, Intermediate tendency = 10–90th MSWEadj percentile, Evening tendency = Latest 10th MSWEadj percentile. %E = percentage from total energy intake. MSWEadj = Midsleep on weekends, adjusted for possible sleep debt on weekdays.

Model 1—adjusted for age and sex (and sleep duration for MSWEadj and chronotype tendency). Model 2—adjusted for variables in Model 1 + age and sex-adjusted body mass index, daylight length, single adult household, and physical activity. Values in italics indicate significance with Benjamini and Hochberg adjusted *p*-values and * indicates the level of significance (* = adj. *p* < .05).

#### Chronotype tendency as morning, intermediate, and evening tendency type.

Relative to having an intermediate chronotype tendency, having a morning chronotype tendency was not associated with any food, energy, or nutrient intake (Table 2). Compared to having intermediate chronotype tendency, an evening type was associated with less healthy diet components, such as lower vegetable consumption (non-transformed B-estimate: −2.49 g/MJ, 95% CI: [−4.79, −0.20], *p* = .03) and higher consumption of sugary foods (non-transformed B-estimate: 4.74 g/MJ, 95% CI: [2.40, 7.07], *p* < .0001). Likewise, compared to intermediate tendency chronotypes, having an evening tendency was significantly associated with 1.43 (95% CI: [0.32, 2.54], *p* = .01) greater %E intake of added sugar (Table 3). Approximately 2.3%–4.5% of the variance in dietary intake was explained by all fully adjusted models.

#### Sleep duration, sleep variability, and social jetlag.

Neither sleep duration, variability in sleep duration and midpoint, nor social jetlag was significantly associated with food consumption (Table 2), total energy intake, or macronutrient composition of the reported diet (Table 3, Supplementary Table 1).

## Discussion

This study found preschool-aged children with an evening chronotype tendency to have poorer sleep habits (e.g. greater social jetlag and day-to-day sleep variability) than morning and intermediate chronotypes. As hypothesized, an evening chronotype tendency was associated with less healthy dietary habits, such as lower vegetable consumption and greater added sugar and sugary food consumption, compared to intermediate types. In addition to slightly lower vegetable and higher sugary food consumption, a later chronotype (assessed as continuous MSWEadj) was also associated with lower fiber intake. However, this study found no associations with children’s habitual sleep dimensions or chronotype with energy intake or macronutrient diet composition.

This study provides novel insight into associations between sleep and diet among 3- to 6-year-olds, which have been scarcely studied with respect to chronotype, social jetlag, or variability in sleep habits. Findings from this study suggest that the associations observed between later chronotype and less healthy dietary habits in adolescents and adults [26, 45] may begin much earlier in life than expected. Though no previous study on preschool-aged children has investigated associations between earlier and later chronotypes with diet, one study has found similar results when examining associations between the midpoint of sleep from parent-reported sleep habits and diet. The study on 354 low-income preschoolers in the United States found that later weekday and weekend sleep midpoints were associated with unfavorable dietary patterns, such as those low in vegetables, legumes, and fish or high in processed and fried foods [33]. An experimental study restricting 10 preschoolers’ bedtimes for approximately 3 hours found increases in energy (21%), sugar (25%), and carbohydrate (14%) intake the next day with persisting increased energy intake (14%) also on the recovery day [46]. The findings from the relatively small trial indicate that an acute delay in bedtime, causing sleep loss, can unfavorably affect diet in young children. Though no associations with habitual sleep duration or reported diet were observed in our study, having an evening chronotype tendency was also associated with higher added sugar intake and sugary food consumption, as well as lower vegetable consumption than children with an intermediate chronotype tendency. In line with our findings, shorter sleep duration was not associated with increased weight status among DAGIS participants in a previous study [37]. However, a diet persistently higher in sugary foods and lower in vegetables, which was associated with having a later chronotype in the present study, could diminish metabolic health over time. Therefore, longitudinal studies are needed to fully understand the long-term effects on energy intake and adiposity from the associations between chronotype and higher added sugar intake observed in younger children. This is particularly important to do in early childhood since this is when lifelong food habits are formed and children begin to exert more autonomy in food choices.

Not surprisingly, the current study found children with evening chronotype to have the most social jetlag, which is used as another indicator for circadian misalignment. Yet, somewhat surprisingly, no significant associations were observed between social jetlag and diet. Sleep variability in both duration and midpoint yielded similarly null findings. However, the associations between diet and social jetlag or sleep variability are still relatively unexplored among young children. Existing studies have comparably smaller sample sizes and focus on vulnerable populations, which could amplify associations between habitual sleep and diet [30, 32, 33]. For instance, cross-sectional findings of preschool-aged children from low-income families have found greater social jetlag to be associated with a dietary pattern high in processed and fried foods in a sample of 354 children [33] and higher overall energy intake in a sample of 51 children [32]. Similar to our findings, variability in sleep duration was not associated with either the overall energy intake or the macronutrient composition of the diet among 368 preschoolers predisposed to obesity [30]. However, contrary to our findings, variability in sleep duration was reported to be associated with higher consumption of SSB and added sugar, as well as lower consumption of fruits and vegetables [30]. When comparing findings, it is important to consider that our study population exhibited higher socioeconomic status than national averages, as indicated by parental education level, which in turn may have attenuated the associations observed in our study. Specifically, families in our study population were more highly educated (37% reported the highest level of education in the family being a master’s degree or higher) than national averages of similarly aged adults at the time of the study in Finland (roughly 20%, respectively) [47]. Nevertheless, we observed that children with a tendency toward evening chronotype had greater social jetlag and sleep variability compared to morning and intermediate chronotype tendencies. These findings highlight the importance of considering social jetlag and sleep variability in understanding the early stages of circadian rhythm disruption and its potential long-term consequences, such as increased adiposity, even during preschool years.

Regarding sleep duration, insufficient sleep duration has been found to be associated with a less healthy diet in early childhood [22]. However, similar to our null findings, a study on 2- to 6-year-olds reported no associations between sleep duration and added sugar, SSB, fruit and vegetable, or macronutrient intakes [30]. Among preschool-aged children, there is no convincing evidence that sleep duration and energy intake are inversely associated as most [32, 48, 49], though not all [30], studies show null associations.

Despite supporting literature, our cross-sectional findings do not provide clarity on whether sleep–wake rhythms in children affect dietary behaviors or vice versa. However, the relationships between sleep and diet are recognized as bidirectional [22, 50]. Food intake is also considered a zeitgeber that can contribute to the entrainment of endogenous sleep–wake rhythms [51]. Therefore, additional research is needed to uncover how the timing, composition, and amount of food consumed near bedtime may influence sleep in younger children. Though notable, it is still important to refrain from overemphasizing the effect of the associations between sleep and dietary intake. It was neither observed nor expected that a highly multifactorial behavior, such as food consumption, would be largely explained by sleep, given its complex nature. Moreover, there is a possibility that the associations observed are attributable to confounding factors that were not accounted for in this study, such as daytime sleep or sleeping in a shared bedroom. Though approximately 70% of children in 2015 aged 1–6 years in Finland attended municipal-provided preschool [52], the study population is not representative of children fully in home care. Likewise, the sample may not be representative of the whole country since only municipalities from western and southern Finland were invited to participate in the study. Ideally, longitudinal studies should aim to clarify the relationship between habitual sleep during childhood, chronotype, diet, and adiposity over time.

The current study used phenotypical expression of objectively measured weekend sleep–wake behavior as an indicator for chronotype instead of circadian preference questionnaires. The chronotype tendency percentiles from MSWEadj (upper and lower 10th percentile) were conservatively demarcated based on the lowest prevalence of evening chronotype identified from questionnaires in previous studies of this age group [6–9]. Still, our classification only describes the most extreme morning and evening tendencies in relation to the DAGIS study population, which may not reflect the true preferences or distribution of chronotype in preschool-aged children. Additionally, our findings suggest that the available daylight length may have a role in circadian phenotype, which has the potential to increase the risk for circadian misalignment among children with a later chronotype during seasons with less sunlight. Future studies should also include light exposure measures and define chronotypes more objectively with validated measures and biomarkers, such as dim light melatonin onset, cortisol levels, or 24-hour activity acrophases. More explorative studies on chronotype in similarly aged children are needed to reliably describe chronotype based on tendency of sleep timing.

In addition, an essential component of measuring chronotype from the midpoint of sleep is to assess free days with adjustment for sleep debt collected on days with work or school [3]. A limitation of this study included weekends being treated as free days, assuming that children can sleep and wake up without external influence. Social obligations that alter sleep timing could hinder weekends from being truly “free” in terms of sleep behavior. Moreover, it could be that the sleep dimensions observed may be more reflective of co-habiting parental or sibling preferences and schedules. Until children reach an age of self-sufficiency, sleep timing is likely codependent on their caregivers’ work and social schedules, which should be considered in future assessments.

Alongside the above-mentioned limitations, this study has many strengths. Various dimensions of habitual, objectively collected sleep were examined, including duration, variability in midpoint and duration, social jetlag, and chronotype based on MSWEadj as well as tendency towards morning, intermediate, or evening chronotype. The sample was relatively large compared to similar studies in preschool-aged children [30, 32, 33, 48, 49] and the compliance rate for wearing the ActiGraph was fairly high; most children in the study had data for seven nights of sleep. ActiGraph hip placement may have helped increase compliance but at a potential cost to estimation accuracy. Sleep assessment may be overestimated with hip-worn actigraphy because a device placed on the trunk of the body is less sensitive to small movements compared to the wrist. Reassuringly, however, actigraphy-estimated sleep duration was not overestimated compared to parent reports in this study population [37]. The 3-day diet record allowed for more detailed analysis of energy, nutrient, and food intake compared to a food frequency questionnaire. However, similar to self-reported intake, proxy-reported intake is unavoidably vulnerable to recall and social desirability bias. Lastly, many demographic and behavioral characteristics were assessed as potential covariates in the analyses.

## Conclusion

This study was the first to explore demographic and behavioral differences among morning, intermediate, and evening chronotype tendencies in preschool-aged children. Children identified as having an evening chronotype tendency had greater social jetlag and sleep variability as well as less healthy reported dietary habits, with higher added sugars and sugary foods consumption and lower vegetable consumption than children with intermediate chronotype tendencies. Having a later chronotype (MSWEadj) was similarly associated with higher sugary food and added sugar intake as well as lower vegetable and fiber intake. The associations mirror findings from studies on older children and adults, suggesting that the negative effects of a later chronotype, including circadian misalignment, can begin to accumulate at a remarkably young age. Public health efforts should be aimed toward awareness of the development of chronic circadian misalignment in children and intervention should be targeted to at-risk children (i.e. children with affinity toward evening chronotypes) to better improve sleep and dietary habits.

## Supplementary Material

zpae026_suppl_Supplementary_Tables

## Data Availability

The data underlying this article will be shared on reasonable request to the research group principal investigator (ER).
