# Supplementary material for: A tendency toward evening chronotype associates with less healthy diet among preschoolers: cross-sectional findings from the DAGIS study
Source: Sleep Adv. 2024 Apr 20;5(1):zpae026. doi: 10.1093/sleepadvances/zpae026 (PMC11085840; doi:10.1093/sleepadvances/zpae026)
Supplement: zpae026_suppl_Supplementary_Tables [file zpae026_suppl_supplementary_tables.docx]

**Supplemental Table 1.** Multivariate Linear Regression Analyses of Sleep and Chronotype Tendency as Predictors of Saturated, Polyunsaturated, and Monounsaturated Fatty Acid Intake in DAGIS Study Participants (n=636)

|  | Saturated fatty acid  (%E) | | | Monounsaturated fatty acid  (%E) | | | Polyunsaturated fatty acid  (%E) | | |
| --- | --- | --- | --- | --- | --- | --- | --- | --- | --- |
|  | Crude Model | Model 1  B (95% CI) | Model 2  B (95% CI) | Crude model | Model 1  B (95% CI) | Model 2  B (95% CI) | Crude model | Model 1  B (95% CI) | Model 2  B (95% CI) |
| MSWEadj | 0.14  (-0.15, 0.43) | 0.10  (-0.19, 0.40) | 0.001  (-0.33, 0.32) | 0.09  (-0.13, 0.30) | 0.08  (-0.13, 0.30) | 0.07  (-0.17, 0.31) | -0.04  (-0.18, 0.10) | -0.05  (-0.19, 0.09) | -0.03  (-0.18, 0.12) |
| Chronotype tendency^1^ |  |  |  |  |  |  |  |  |  |
| Intermediate | ref | ref | ref | ref | ref | ref | ref | ref | ref |
| Morning | -0.40  (-1.07, 0.27) | -0.33  (-1.01, 0.34) | -0.23  (-0.97, 0.51) | -0.28  (-0.77, 0.21) | -0.27  (-0.76, 0.23) | -0.22  (-0.75, 0.32) | 0.07  (-0.39, 0.25) | 0.05  (-0.37, 0.27) | -0.08  (-0.42, 0.27) |
| Evening | -0.01  (-0.68, 0.66) | -0.05  (-0.73, 0.62) | -0.01  (-0.73, 0.72) | -0.09  (-0.57, 0.40) | -0.09  (-0.58, 0.41) | -0.02  (-0.51, 0.55) | -0.11  (-0.43, 0.21) | -0.12  (-0.44, 0.20) | -0.04  (-0.38, 0.30) |
| Sleep duration | -0.14  (-0.53, 0.24) | -0.14  (-0.53, 0.16) | -0.27  (-0.74, 0.21) | -0.02  (-0.30, 0.26) | -0.01  (-0.29, 0.27) | -0.03  (-0.38, 0.31) | -0.05  (-0.24, 0.13) | -0.04  (-0.22, 0.15) | -0.004  (-0.23, 0.22) |
| Social jetlag | -0.03  (-0.48, 0.41) | -0.07  (-0.51, 0.38) | -0.27  (-0.76, 0.23) | 0.11  (-0.21, 0.44) | 0.11  (-0.21, 0.44) | 0.02  (-0.34, 0.37) | 0.03  (-0.19, 0.24) | 0.02  (-0.19, 0.24) | -0.01  (-0.22, 0.24) |
| Variability in sleep duration | 0.62  (-0.09, 1.33) | 0.62  (-0.10, 1.33) | 0.57  (-0.24, 1.39) | 0.47  (-0.05, 0.99) | 0.49  (-0.03, 1.02) | 0.45  (-0.14, 1.04) | -0.02  (-0.36, 0.32) | 0.01  (-0.33, 0.35) | 0.01  (-0.37, 0.39) |
| Variability in sleep midpoint | 0.28  (-0.84, 1.39) | 0.26  (-0.86, 1.38) | 0.12  (-1.14, 1.38) | 0.36  (-0.47, 1.16) | 0.37  (-0.45, 1.19) | 0.33  (0.57, 1.25) | 0.02  (-0.51, 0.56) | 0.05  (-0.48, 0.59) | 0.03  (-0.56, 0.62) |

1. Morning tendency = Earliest 10th MSWEadj percentile, Intermediate tendency = 10-90^th^ MSWEadj percentile, Evening tendency = Latest 10th MSWEadj percentile.

%E = percentage from total energy intake.

MSWEadj = Midsleep on weekends, adjusted for possible sleep debt on weekdays.

Model 1 – adjusted for age and sex (and sleep duration for MSWEadj and chronotype tendency)

Model 2 – adjusted for variables in Model 1 + daylight length, single adult household, and physical activity.

**Supplemental Table 2.** Multivariate Linear Regression Analyses of Sleep and Chronotype Tendency as Predictors of Food Consumption in DAGIS Study Participants (n=636)

|  | Vegetable^1^  (g/MJ) | Fruit^1^  (g/MJ) | Sugary foods^1^  (g/MJ) | Sugar sweetened beverages^1^ (g/MJ) |
| --- | --- | --- | --- | --- |
|  | Crude Model  B (95% CI) | Crude Model  B (95% CI) | Crude Model  B (95% CI) | Crude Model  B (95% CI) |
| MSWEadj | *-0.17**  *(-0.30, -0.04)* | -0.01  (-0.21, 0.18) | *0.36****  *(0.21, 0.51)* | 0.18  (-0.03, 0.38) |
| Chronotype tendency^2^ |  |  |  |  |
| Intermediate | ref | ref | ref | ref |
| Morning | 0.17  (-0.13, 0.47) | 0.07  (-0.38, 0.52) | *-0.38**  *(-0.73, -0.04)* | -0.04  (-0.51, 0.44) |
| Evening | *-0.37**  *(-0.68, -0.07)* | 0.29  (-0.16, 0.74) | *0.61***  *(0.27, 0.96)* | 0.41  (-0.06, 0.89) |
| Sleep duration | 0.14  (-0.04, 0.31) | 0.23  (-0.03, 0.49) | -0.05  (-0.25, 0.15) | -0.003  (-0.28, 0.27) |
| Social jetlag | -0.09  (-0.29, 0.11) | -0.07  (-0.37, 0.23) | 0.22 (-0.01, 0.46) | 0.16  (-0.15, 0.48) |
| Variability in sleep duration | -0.14  (-0.46, 0.18) | 0.36  (-0.12, 0.84) | 0.09  (-0.29, 0.46) | 0.24  (-0.26, 0.75) |
| Variability in sleep midpoint | -0.21  (-0.71, 0.30) | 0.09  (-0.66, 0.85) | 0.28  (-0.31, 0.86) | 0.44  (-0.35, 1.23) |

1. Variables square root transformed. 2. Morning tendency = Earliest 10th MSWEadj percentile, Intermediate tendency = 10-90^th^ MSWEadj percentile, Evening tendency = Latest 10th MSWEadj percentile.

MSWEadj = Midsleep on weekends, adjusted for possible sleep debt on weekdays.

Values in italics indicate significance with Benjamini and Hochberg adjusted p-values and * indicates the level of significance (*= adj. p<0.05, ** = adj. p<0.01, ***= adj. p<0.001).

**Supplemental Table 3.** Multivariate Linear Regression Analyses of Sleep and Chronotype Tendency as Predictors of Energy and Nutrient Intake in DAGIS Study Participants (n=636)

|  | Energy  (MJ) | Carbohydrate  (%E) | Protein  (%E) | Fat  (%E) | Added sugar  (%E) | Fiber  (g/MJ) |
| --- | --- | --- | --- | --- | --- | --- |
|  | Crude Model  B (95% CI) | Crude Model  B (95% CI) | Crude Model  B (95% CI) | Crude Model  B (95% CI) | Crude Model  B (95% CI) | Crude Model  B (95% CI) |
| MSWEadj | *-0.16**  *(-0.29, -0.04)* | -0.04  (-0.59, 0.50) | -0.10  (-0.36, 0.15) | 0.24  (-0.28, 0.77) | 0.52  (0.06, 0.98) | *-0.09**  *(-0.16, -0.02)* |
| Chronotype tendency^1^ |  |  |  |  |  |  |
| Intermediate | ref | ref | ref | ref | ref | Ref |
| Morning | -0.07  (-0.36, 0.22) | 0.84  (-0.41, 2.09) | 0.01  (-0.58, 0.60) | -0.95  (-2.15, 0.26) | -0.07  (-0.99, 1.13) | 0.01  (-0.16, 0.15) |
| Evening | -0.26  (-0.55, 0.04) | 0.89  (-0.36, 2.14) | -0.58  (-1.17, 0.003) | -0.15  (-1.36, 1.05) | *1.57**  *(0.51, 2.63)* | -0.13  (-0.29, 0.02) |
| Sleep duration | -0.10  (-0.26, -0.07) | 0.20  (-0.52, 0.92) | 0.07  (-0.41, 0.27) | -0.22  (-0.91, 0.47) | -0.11  (-0.72, 0.51) | 0.06  (-0.03, 0.15) |
| Social jetlag | -0.15  (-0.34, 0.04) | -0.15  (-0.98, 0.68) | 0.04  (-0.35, 0.43) | 0.17  (-0.63, 0.97) | 0.13  (-0.58, 0.84) | -0.07  (-0.18, 0.03) |
| Variability in sleep duration | *-0.44**  *(-0.75, -0.13)* | -0.77  (-2.10, 0.57) | -0.47  (-1.09, 0.16) | 1.42  (0.14, 2.70) | 0.20  (-0.94, 1.34) | -0.02  (-0.19, 0.15) |
| Variability in sleep midpoint | *-0.73**  *(-1.23, -0.25)* | -0.18  (-2.27, 1.91) | -0.41  (-1.39, 0.56) | 0.92  (-1.08, 2.94) | 1.14  (-0.64, 2.92) | -0.19  (-0.45, 0.08) |

1. Morning tendency = Earliest 10th MSWEadj percentile, Intermediate tendency = 10-90^th^ MSWEadj percentile, Evening tendency = Latest 10th MSWEadj percentile.

%E = percentage from total energy intake.

MSWEadj = Midsleep on weekends, adjusted for possible sleep debt on weekdays.

Values in italics indicate significance with Benjamini and Hochberg adjusted p-values and * indicates the level of significance (*= adj. p <0.05, ** = adj. p <0.01, ***= adj. p <0.001).
